# Morphological Features and *In Vitro* Cytopathic Effect of *Acanthamoeba griffini* Trophozoites Isolated from a Clinical Case

**DOI:** 10.1155/2014/256310

**Published:** 2014-09-08

**Authors:** Arturo González-Robles, Lizbeth Salazar-Villatoro, Maritza Omaña-Molina, Maria Reyes-Batlle, Carmen M. Martín-Navarro, Jacob Lorenzo-Morales

**Affiliations:** ^1^Department of Infectomics and Molecular Pathogenesis, Center for Research and Advanced Studies, CINVESTAV-IPN, Avenida Instituto Politécnico Nacional 2508, San Pedro Zacatenco, 07360 Mexico, DF, Mexico; ^2^Faculty of Superior Studies, Biology, UNAM, Los Reyes Iztacala, 54090 Tlalnepantla, MEX, Mexico; ^3^University Institute of Tropical Diseases and Public Health of The Canary Islands, University of La Laguna, Avenida Astrofísico Francisco Sánchez SN, San Cristóbal de La Laguna, Tenerife, 38203 Canary Islands, Spain; ^4^Centre for Integrative Physiology, School of Biomedical Sciences, University of Edinburgh, Hugh Robson Building, George Square, Edinburgh EH8 9XD, UK

## Abstract

Light and transmission electron microscopy observations are reported on the structure and *in vitro* cytopathic effect of *Acanthamoeba griffini* trophozoites isolated from a clinical case. Live trophozoites were moderately active with a remarkable pleomorphism which changed from ovoid to quite elongated shapes. When moving, amoebae formed cytoplasmic projections such as wide lamellae and acanthopodia of diverse size and thickness which contain a significant amount of actin. Ultrastructurally, the cytoplasm showed the main organelles found in other free-living amoebae. Coincubation of trophozoites with MDCK cell monolayers resulted in a local damage to target cells after 24 h of interaction, suggesting that the cytopathic effect is contact-dependent. By transmission electron microscopy, amoebae appeared to engulf small portions of the MDCK cells; however, the cells that were not in contact with trophozoites had an unaltered morphology. When epithelial monolayers were incubated with conditioned medium for 24 h, small areas of cell injury were also observed. The phylogenetical analysis as well as the sequencing of the acquired amplified product for the DF3 region of the amoebae isolate confirmed that it belongs to genotype T3, which includes other pathogenic amoebae; besides the activity of two drugs currently used against *Acanthamoeba* was tested on *A. griffini*.

## 1. Introduction

Free-living amoebae from the genus* Acanthamoeba *are commonly found in soil and aquatic environments worldwide [1–3]. These amoebae have been isolated from very diverse habitats, including water from the Antarctic [4], bottled water [5], swimming pools [6], dental units [7], eye wash stations [8], and even from dust in the atmosphere [9].

*Acanthamoeba* spp. are the most common and opportunistic amphizoic protozoa.* Acanthamoeba castellanii* is one of the etiological agents of chronic granulomatous amebic encephalitis [10] and amoebic keratitis, a progressive and painful sightthreatening eye infection [11–13]. The first description of* Acanthamoeba griffini* was done by Sawyer [14], but reports on this amoeba on the scientific literature are very limited. Molecular analyses performed in 2003 [15, 16] concluded that this amoeba belongs to the T3 genotype, which is clinically relevant since other pathogenic amoebae are also included in this cluster [17].

By means of light and transmission electron microscopy we present some observations on the morphology of this amoeba isolated from a case of keratitis as well as its cytopathic effect on MDCK epithelial cell monolayers.

## 2. Material and Methods

### 2.1. Amoebae

Amoebae were isolated in October 2013 from a severe case of keratitis from both a contact lens and a corneal scrape (Association to prevent Blindness in Mexico, Luis Sánchez Bulnes Hospital, Mexico City, Mexico). After axenization, it was cryopreserved immediately.

### 2.2. Isolation and Maintenance of* Acanthamoeba* sp. in Monoxenic Cultures

The technique used for the recovery and maintenance of* Acanthamoeba* sp. from clinical and environmental sources has been described elsewhere [18, 19]. Briefly, primary isolation was performed by using 1.5% nonnutrient agar plates seeded with heat-killed* Enterobacter aerogenes*. Clinical samples were then streaked on the agar. Subsequent incubation was performed at room temperature (22–24°C) for 10 days. Upon evidence of growth, cultures were established by transference of a single double-walled cyst to fresh agar medium.

### 2.3. Axenic Cultures

Monoxenic cultures were selected from areas of profuse amoebic growth. Selected pieces of agar were transferred to axenic culture media such as phosphate-biotriptase-serum glucose medium (PBSGM) and 2% Bacto Casitone (pancreatic digest of casein, Becton-Dickinson, Sparks, MD) medium (DIFCO), which are culture media widely used for growth and amoebic development [9, 20]. Both media were supplemented with 10% fetal bovine serum (Equitech-bio, Kerville, Tex USA) and 1% antibiotics (penicillin, 100 mg/mL; streptomycin, 10 mg/mL). Trophozoites were incubated in both media at 30°C in borosilicate tubes (Pyrex, Mexico), and the medium was changed twice daily for two days and thereafter once daily for three more days. The cultures were considered axenic if no bacterial growth was observed.

Axenized trophozoites were grown and maintained in axenic culture in 2% Bacto Casitone supplemented with 10% fetal bovine serum (Equitech-bio, Kerville, Tex USA) and 1% antibiotics. Cultures were incubated at 30°C and trophozoites were harvested at the end of the logarithmic phase of growth.

### 2.4. Temperature Tolerance Test

To determine the optimal culture medium and temperature for growth, amoebae were incubated at 25, 30, and 37°C in borosilicate tubes (Pyrex, Mexico). Optimal growth was determined by plotting logarithmic growth phase curves (assays were performed in triplicate). The viability of the trophozoites was determined by trypan blue (0.4%) exclusion.

### 2.5. Pathogenicity Test

In order to induce granulomatous amoebic encephalitis as a method to evaluate the virulence of the amoebae isolate, intranasal instillation was used in a mouse model of infection. Briefly, axenic cultures of* A. griffini* trophozoites in the exponential phase of growth (72 h) were chilled at 4°C and concentrated by centrifugation for 5 min at 2500 rpm. 2 × 10^5^ trophozoites were resuspended in 200 *μ*L of fresh culture medium or isotonic solution and instillated into the nostrils of 5 male Balb/C mice [21]. A group of five mice was inoculated with culture medium without amoebae and used as controls. Mice were sacrificed 21 days after amoebae inoculation. The brain, liver, lungs, and kidneys from sacrificed animals were cultured on agar plates with nonnutritive enriched medium (NNE) to retrieve the amoebae. Experimental animals were maintained in optimal conditions according to international standards that regulate the care and management of experimental animals.

### 2.6. Drugs Activity Assays

The activity of two drugs currently used against* Acanthamoeba* was tested on* Acanthamoeba griffini*. Chlorhexidine (Chlorhexidine digluconate, Alfa Aesar, Germany) is a standard antiseptic belonging to the biguanides family which are commonly used in contact lens maintenance solutions; and voriconazole (Sigma, Madrid, Spain) an inhibitor of ergosterol synthesis that has been proven previously to be highly effective against clinical strains of* Acanthamoeba* [22, 23]. For the sensitivity and activity assays, a type strain from the American Type Culture Collection (ATCC),* Acanthamoeba castellanii* Neff ATCC 30010, genotype T4 was used as a control.

For the activity assays a previously developed colorimetric 96-well microtiter plate assay, based on the oxide-reduction of Alamar Blue assay [24], was used for the determination of drug efficacy against the trophozoites of the selected* Acanthamoeba* strains. Subsequently the plates were analyzed, over a period from 72 to 120 h, on a Microplate Reader Model 680 (Biorad, Hercules, CA) using a test wavelength of 570 nm and a reference wavelength of 630 nm. For those strains that were sensitive to the assayed drugs, the percentage of inhibition and 50% inhibitory concentrations (IC_50_) were calculated by linear regression analysis using a 95% confidence limit. All experiments were performed three times each in duplicate and the mean values were also calculated. A paired two-tailed *t*-test was used for analysis of the data. Values of *P* < 0.05 were considered significant. The inhibition curves of the statistical analysis were developed using the Sigma Plot 12.0 software programme (Systat Software Inc.).

### 2.7. Culture of MDCK Cells

Monolayers of epithelial cells of the established MDCK line of canine kidney origin (Madin Darby Canine Kidney) were grown on 25 cm^2^ cell culture flasks (Corning Incorporated, NY) in Dulbecco's modified Eagle's medium (Gibco, Grand Islands, NY) supplemented with 10% fetal bovine serum (Equitech-bio, Kerville, Tex USA) and antibiotics in a 5% CO^2^ atmosphere at 37°C.

### 2.8. Coincubation of Trophozoites with MDCK Cells

MDCK cell monolayers were trypsinized and grown in round plastic cover slips placed in 24 well styrene plates. Cultures were maintained at 37°C in a 5% CO^2^ atmosphere, and 24 h later confluent monolayers were obtained. Trophozoites were added in a 1 : 2 target cell : amoeba ratio in a mixture of equal proportions of Bacto Casitone and Dulbecco's modified Eagle's medium (Gibco BRL). Incubations were carried out for different times (6, 12, 16, and 24 h) under the same conditions.

### 2.9. Incubation of MDCK Monolayers with Conditioned Medium

The conditioned medium was obtained as follows: 6 × 10^6^ trophozoites from a culture in the exponential phase of growth were placed in culture flasks containing 7 mL of fresh Bacto Casitone-DMEM serum-free medium (1 : 1) and incubated at 30°C for 24 h. Trophozoites were chilled on ice for 10 min and centrifuged for 5 min at 2500 rpm. The supernatant was removed, centrifuged, and filtered through a 0.22 *μ*m membrane (Millipore, Bedford, Massachusetts). MDCK cell monolayers were incubated for 24 h with a mixture of conditioned medium and Dulbecco's modified Eagle's medium (Gibco BRL) in equal proportions.

### 2.10. Confocal Microscopy

Amoebae cultured for 72 h were chilled in an ice-water mixture for 5 min, pelleted by centrifugation, and fixed with 4% paraformaldehyde for 1 h. Samples were then washed with Dulbecco's Phosphate Buffered Saline (DPBS) and blocked for 1 h with 10% fetal bovine serum diluted in DPBS. Afterwards, the cells were washed with DPBS, treated with a 1 : 25 solution of phalloidin-tagged rhodamine complex (Molecular Probes, Eugene, OR, USA) for 20 min at 37°C, and washed exhaustively with DPBS. Samples were mounted with Vectashield (Vector Laboratories Inc., Burlingame, CA, USA) and observed in a LS 700 Laser Scanning Microscope (Carl Zeiss GmbH, Germany).

### 2.11. Light Microscopy

Observations of live and fixed trophozoites and their interaction with MDCK monolayers were performed using a Zeiss Axiophot photomicroscope equipped with an AxioCam MRc digital camera (Carl Zeiss GmbH, Germany).

### 2.12. Electron Microscopy

#### 2.12.1. Transmission Electron Microscopy

Samples were fixed at room temperature with 2.5% glutaraldehyde in 0.1 M sodium cacodylate buffer, pH 7.2, postfixed with 1% osmium tetroxide in the same buffer, dehydrated in increasing concentrations of ethanol, and embedded in epoxy resins. Thin sections were observed in a JEOL JEM-1011 transmission electron microscope (JEOL Ltd. Tokyo, Japan).

#### 2.12.2. Scanning Electron Microscopy

Samples were fixed with 2.5% glutaraldehyde in 0.1 M sodium cacodylate buffer pH 7.2, dehydrated with increasing concentrations of ethanol, critical-point dried (31°C and 1100 psi) using a Samdri apparatus (Tousimis Corp., Rockville. MD), and coated with gold particles in an ion sputtering device (JEOL JFC-1100). Samples were then examined with a JEOL-JSM 7100 F scanning electron microscope (JEOL Ltd. Tokyo Japan).

### 2.13. DNA Extraction and Genotyping of Isolates

*Axenic Isolates.* Fungi and bacteria-free plates were transferred to axenic culture by placing the amoebae in PYG medium as previously described for further morphological and molecular analyses [17, 25, 26]. Amoebae were grown exponentially (10^6^ cells/mL) for DNA extraction and activity assays.

DNA from amoebic cultures was extracted by placing 1-2 mL of axenic* Acanthamoeba* cultures directly into the Maxwell 16 Tissue DNA Purification Kit sample cartridge.* Acanthamoeba* genomic DNA was purified using the Maxwell 16 Instrument as described in the Maxwell 16 DNA Purification Kits Technical Manual number TM284. DNA yield and purity were determined using the NanoDrop spectrophotometer.

rRNA gene amplifications (DF3 region) were also performed as previously described [26, 27]. PCR products were purified using the Qiaquick PCR purification kit (Qiagen, Hilden, Germany) and sequenced using a MEGABACE 1000 automatic sequencer (Healthcare Biosciences, Barcelona, Spain) in the University of La Laguna Sequencing Services (Servicio de Secuenciación SEGAI, University of La Laguna). The obtained sequences were aligned using Mega 5.0 software program [28]. Phylogenetic analyses were carried out using maximum parsimony, minimum evolution, and maximum likelihood optimality criteria, implemented in Mega 5.0 [28]. Transition : transversion ratios were estimated by maximum likelihood heuristic searches. Estimates of node support were obtained by performing 1000 bootstrap replicates. Genotype identification was based on sequence analysis of DF3 region as previously described by comparison with the available* Acanthamoeba* DNA sequences in Genbank database [26, 29].* Acanthamoeba castellanii* Neff ATCC 30010 DNA was used as a positive control in the PCR reactions. Diagnostic Fragment 3 sequence for the new isolate is deposited in the Genbank database under the accession number: KF914142.

## 3. Results

### 3.1. Light Microscopy

**Figure 1:**
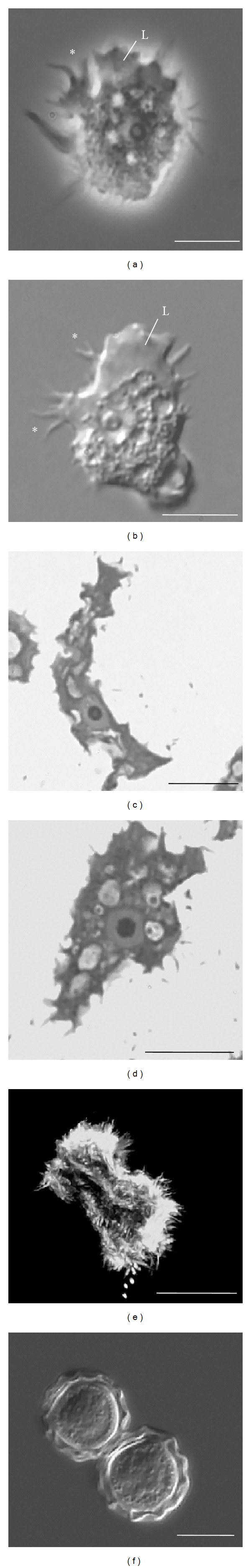
Light and confocal microscopy of* Acanthamoeba griffini* trophozoites. Phase contrast (a) and differential interference-contrast (b) microscopy of a live* A. griffini* trophozoite exhibiting an irregular morphology. A large lamellipodium (L) of smooth appearance as well as several thin and slender acanthopodia that are frequently bifurcated ( *) are seen. (c) and (d) Toluidine blue stained thick resin sections of trophozoites. The morphology of the trophozoites is very diverse, ranging from slender to oval. In both amoebae a single large nucleus along with a round dark nucleolus and numerous vacuoles with diverse content are observed. (e) A trophozoite observed by confocal microscopy after treatment with phalloidin-rhodamine complex exhibiting a positive strong reaction to actin. This protein is located in cytoplasmic projections but it is also detected all around the cell body. (f) Differential interference-contrast image of* A. griffini* cysts with showing clearly defined thick wavy layers located near the rounded endocyst.

Live trophozoites (averaging 25–35 *μ*m) were extremely pleomorphic, moderately mobile and during displacement they exhibited both large lamellae and cytoplasmic acantopodia of varying size and shape. Acantopodia were composed of hyaline cytoplasm of smooth appearance, and many cellular projections were bifurcated (Figures 1(a) and 1(b)). A large round to oval nucleus with a prominent and centrally located nucleolus composed of condensed dark chromatin (Figures 1(c) and 1(d)) as well as abundant vacuoles of different size and content was easily observed (Figure 1(d)).

The cyst of* A. griffini* had an average diameter of 14 *μ*m and showed the morphological features that distinguish the amoebae belonging to group II [30]. The ectocyst consisted of a thick wavy layer located near the more or less rounded endocyst. Both layers were found close together around most of the circumference of the cyst and small pores were observed on the cyst surface (Figure 1(f)).

### 3.2. Confocal Microscopy

Amoebae treated with a phalloidin-rhodamine complex showed a strong reaction to actin, one of the most important structural proteins of cells. Actin was present abundantly in the cytoplasmic projections, although it was also distributed all around the cell body (Figure 1(e)).

### 3.3. Electron Microscopy

#### 3.3.1. Transmission Electron Microscopy

**Figure 2:**
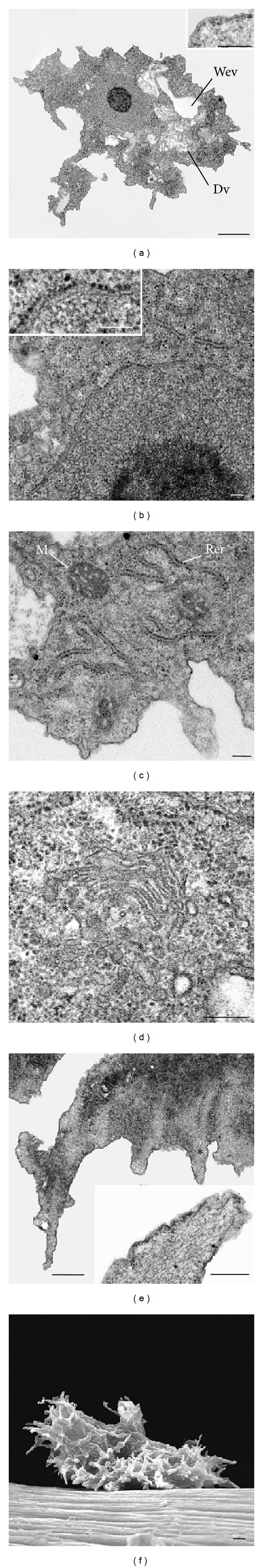
Transmission electron microscopy ((a)–(e)) and scanning electron microscopy (f) of* A. griffini* trophozoites. (a) Low magnification of an amoeba which shows an extremely irregular profile and a large clearly defined granular round nucleus with a circular electron-dense nucleolus. Numerous digestive vacuoles (Dv) with fibrogranular content and a water expulsion vacuole (Wev) are also observed. The plasma membrane presents its classical three-layered structure (insert). Bar = 2 *μ*m. Insert = 0.1 *μ*m. (b) The nuclear envelope is composed of a double membrane with abundant ribosomes on the external side of the envelope. Bar = 0.2 *μ*m. Insert = 0.1 *μ*m. (c) Mitochondria (M) and numerous profiles of rough endoplasmic reticulum (Rer) located near the edge of the cell were clearly defined. Bar = 0.2 *μ*m. (d) Golgi system with various stacked cisternae. Some membrane-bound vesicles were present in the concave area (asterisks). Bar = 0.2 *μ*m. (e) Fibrogranular matrix located in the periphery of a trophozoite. The asterisk indicates a similar region observed at high magnification where abundant actin filaments are observed. Bar = 1.0 *μ*m. Insert = 0.2 *μ*m. (f) A trophozoite in a lateral view is shown in a displacing position with a portion of the cell body facing upwards. The rough and extremely uneven cell surface exhibits abundant cytoplasmic projections, mostly in the manner of filopodia. Bar = 1.0 *μ*m.

As observed by light microscopy, trophozoites seen at low magnification by transmission electron microscopy exhibited an amazing pleomorphism. The cell outline was extremely irregular, ranging from thin and slender acanthopodia to broad lamellae. The cytoplasm presented areas of granular appearance as well as abundant vacuoles with different content most of which corresponded to digestive vacuoles. Occasionally a water expulsion vacuole was observed (Figure 2(a)). The cell membrane was composed of the characteristic trilaminar structure and was approximately 8.0 nm thick (Figure 2(a) insert). This amoeba had a large round to oval nucleus with a well-defined centrally located nucleolus of compact appearance (Figure 2(a)). At higher magnification, the nuclear envelope was clearly visible, composed of a double membrane enclosing a narrow perinuclear space of approximately 0.03 *μ*m. Numerous ribosomes were present on the external side of the envelope (Figure 2(b)). Cytoplasmic organelles such as the rough endoplasmic reticulum and mitochondria were a regular finding (Figure 2(c)). The rough endoplasmic reticulum was clearly defined, usually in close relation with mitochondria and was observed as short tubular segments with arrays of ribosomes on its surface; these structures were frequently located near the edge of the cell. Mitochondria were limited by a double membrane and had an oval to round outline with a dense homogeneous matrix of granular aspect, and some of them presented electron-dense granules. The cristae were observed as tubules of irregular shapes, occasionally branched. In the inner side of the tubules a granular content was present. The Golgi system comprised a roughly saucer-shaped stack of smooth flattened membranous sacs. Some membrane-bound vesicles were observed in the vicinity of the concave side of this system (Figure 2(d)).

When the trophozoites were tagged with a phalloidin-rhodamine complex, a profuse reaction with actin was found in the areas located around the cells profile, particularly in those domains involving cell movement.  Figure 2(e) illustrates a low magnification thin section corresponding to the edge of one trophozoite where the cytoplasm lacks organelles and is constituted by a fibrogranular matrix. At high magnification it was possible to observe abundant actin filaments (Figure 2(e) insert).

#### 3.3.2. Scanning Electron Microscopy

*A. griffini* trophozoites observed by scanning electron microscopy presented a quite uneven rough surface and plenty of cell surface projections such as filopodia of diverse length; sometimes some of them were bifurcated. A common finding was the presence of flat and smooth lamellae, and occasionally amebostomes were also found (Figure 2(f)).

### 3.4. Temperature Tolerance Test

Analysis of growth curves revealed that* A. griffini *grows best from 25°C to 30°C, reaching exponential phase at 72 h, with trophic population and a viability of 100% as determined by trypan blue exclusion. Amoebae incubated at 37°C did not divide. These trophozoites had a round morphology and presented large vacuoles in the cytoplasm and encysted rapidly.

### 3.5. Incubation of MDCK Monolayers with* A. griffini* Trophozoites and Conditioned Medium

**Figure 3:**
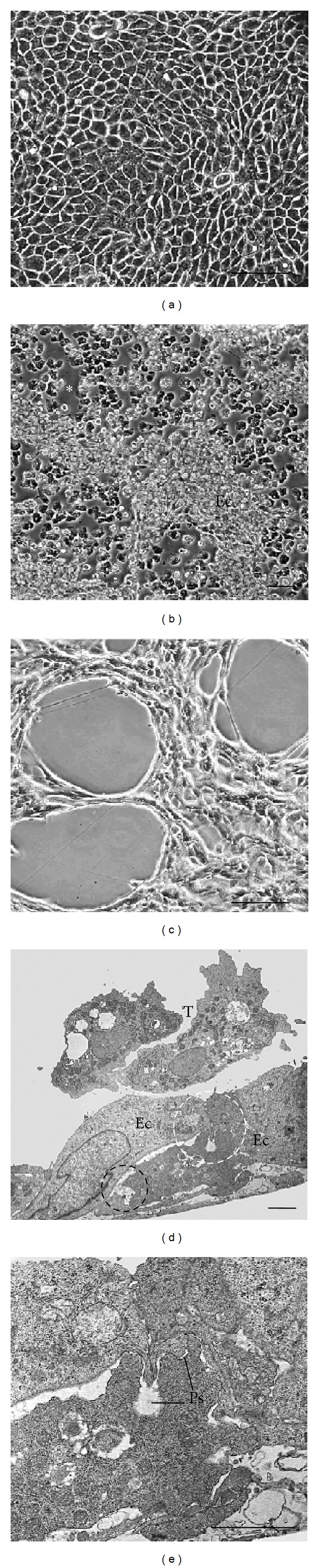
((a)–(c)) Light microscopy analysis of the interaction between* A. griffini* trophozoites and MDCK monolayers. (a) Control of MDCK epithelial cells monolayer incubated for 24 h in a mixture of equal proportions of Bacto Casitone and Dulbecco's modified Eagle's medium. Note the confluent appearance of the monolayer and the regular morphology of the epithelial cells. Bar = 2.0 *μ*m. (b) After 24 h of coincubation between trophozoites and the epithelial cells, a noticeable damage is evident. Some areas of the substrate clearly lacking epithelial cells (asterisk) as well as remaining portions of the altered cell monolayer (Ec) are seen. Bar = 1.0 *μ*m. (c) When MDCK monolayers were incubated with conditioned medium for 24 h rounded areas of varying size missing cells were seen. Bar = 2 *μ*m. ((d),(e)) Thin sections of coincubations between amoebae and MDCK epithelial cells. (d) Trophozoites (T) were found in close contact with the surface of epithelial cells (Ec) and also under the cell layer. Epithelial cells appear undamaged; nevertheless an amoeba is initiating an injury process (white dotted circle) and is also engulfing cell debris (black dotted circle). Bar = 2 *μ*m. (e) High magnification of the previous image where two phagocytic structures (Ps) start to engulf a portion of the epithelial cell. Bar = 2 *μ*m.

Monolayers incubated for 24 h in a mixture of equal proportions of Bacto Casitone and Dulbecco's modified Eagle's medium were structurally well preserved (Figure 3(a)). After 24 h of interaction between* A. griffini* trophozoites and MDCK cell monolayers an evident damage to the epithelial cell monolayer was seen; there were areas of the substrate where MDCK cells were missing and only trophozoites were observed (Figure 3(b)). When epithelial monolayers were incubated with conditioned medium for 24 h, rounded areas of diverse size lacking cells were also seen (Figure 3(c)). In transversal sections observed by transmission electron microscopy, trophozoites were seen in close relation with the apical surface of the cell monolayer; some of them penetrated below the monolayer and detached the epithelial cells, ingesting portions of the cells by means of phagocytic structures of different sizes. The presence of phagocytic structures in the cytoplasm of trophozoites was a regular finding (Figures 3(d) and 3(e)).

### 3.6. Pathogenicity Test

Mice infected with the amoeba showed evidence of illness, as manifested by ruffled fur and aimless wandering, but they were able to recover in few days. Mice were sacrificed 21 days after inoculation. Fragments of the brain, lungs, liver, and kidney were freshly macerated and it was possible to observe only a few trophozoites in the brain and lungs. However, numerous cysts were detected in brain which may be the result of a rapid encystment.

Fragments of extracted organs were cultured at 30°C in agar plates with nonnutritive enriched medium (NNE) to recover the amoebae. Again scarce trophozoites were recovered from brain and lungs and numerous cysts were observed.

### 3.7. Phylogenetic Analysis

**Figure 4:**
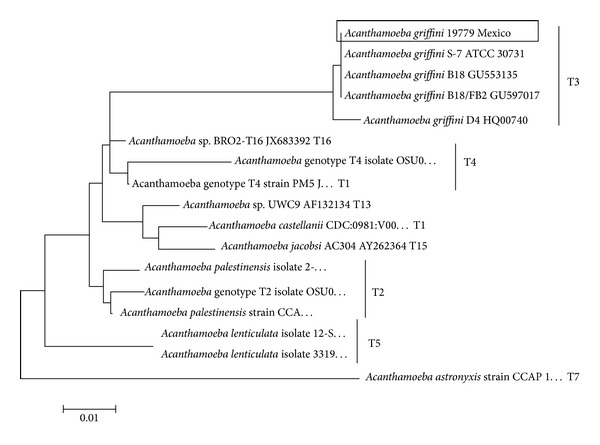
18S rRNA DF3 linearized Neighbour-Joining tree. The phylogenetic analysis was performed using the Kimura two-parameter distance algorithm in MEGA 5.0. The isolate obtained in the present study is identified in the tree (box). The type sequences were taken from GenBank and are presented under the following numbers:* A. astronyxis* strain CCAP 1534/1 genotype T7 Accession number AF239293,* A. castellanii* strain genotype T1 CDC:0981:V006 Accession number U07400,* A. griffini* S-7 ATCC 30731 genotype T3 Accession number U07412,* A. griffini *isolate B18 genotype T3 Accession number GU553135,* A. griffini *isolate B18/FB2 genotype T3 Accession number GU597017,* A. griffini *isolate D4 genotype T3 Accession number HQ00740,* A. lenticulata* isolate 12-SO #KC694184,* A. lenticulata* isolate 33195463 Accession number KC438381,* Acanthamoeba* sp. isolate OSU09-002 Accession number JQ669657,* Acanthamoeba* sp. genotype T2 Isolate OSU09-006 number JQ669661,* Acanthamoeba palestinensis* isolate TW-2 Accession number KC694193,* A. palestinensis* strain CCAP 1547-1 number AF239296,* Acanthamoeba* sp. isolate BRO2-T16 Accession number JX683392,* Acanthamoeba* sp. UWC9 genotype T13 Accession number AF132134,* Acanthamoeba* sp. PM5 genotype T4 Accession number JX494395,* Acanthamoeba *sp. genotype T4 Accession number JQ669660, and* Acanthamoeba jacobsi* AC304 Accession number AY262364.

After sequencing of the obtained amplified product for the DF3 region of the new isolate and performing the phylogenetical analysis, it was concluded that the new isolate belonged to genotype T3 (Figure 4).

### 3.8. Drugs Activity Assays

**Figure 5:**
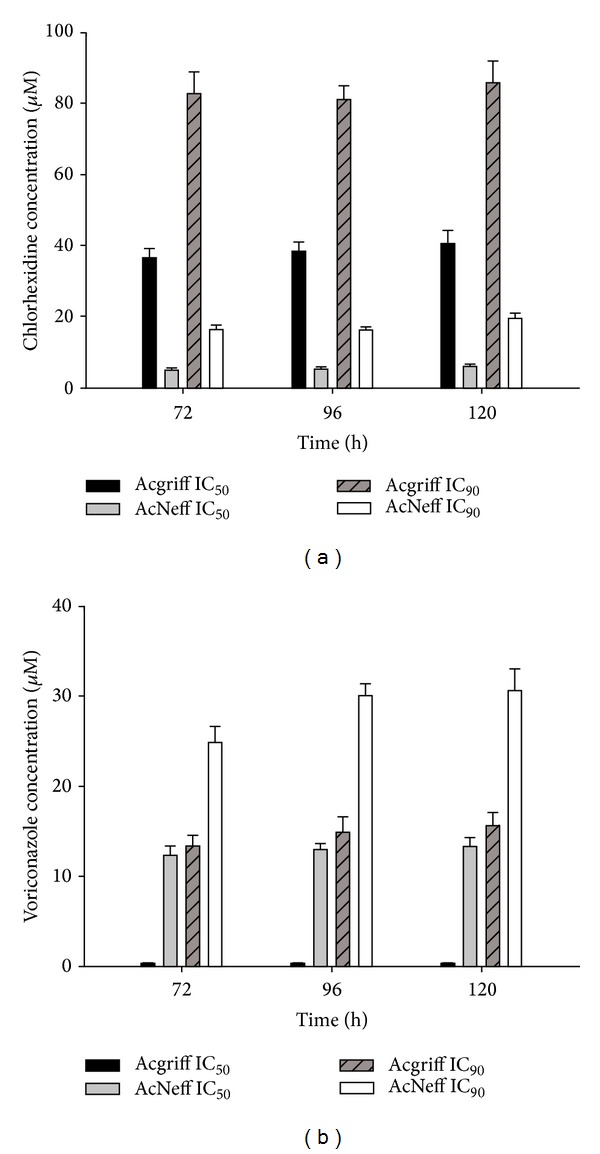
*In vitro *activity of chlorhexidine (a) and voriconazole (b) against the* Acanthamoeba griffini *strain.* Acanthamoeba castellanii* Neff ATCC 30010 was used as a control strain. Activities and concentrations (IC_50_ and IC_90_) of the tested compounds are shown at 72, 96, and 120 h.

All* A. griffini *and* A. castellanii* Neff were sensitive to chlorhexidine and voriconazole assayed microscopically and colorimetrically. Activity assays with chlorhexidine and voriconazole and the IC_50_ data for each strain indicated that both compounds were able to inhibit the growth of these amoebae and in the case of voriconazole, even at low concentrations with IC_90_. However, in this study voriconazole activity was ten times higher than the one observed for chlorhexidine (Figure 5).

## 4. Discussion

In contrast to other free-living amoebas, the morphology of* Acanthamoeba griffini *is extremely variable, ranging from rounded to elongated shapes. The cytoplasm of* A. griffini* trophozoites shows the main cell organelles; a large nucleus with a centrally located electron-dense nucleolus clearly defined profiles of rough endoplasmic reticulum, mitochondria, and a Golgi system as well as vacuoles with diverse content and the presence of abundant actin filaments. Actin is described as one of the most profuse and highly conserved proteins in eukaryotic cells, which is implicated in many vital cellular functions such as cell motility. During cell movement and phagocytosis, prearranged meshes of actin filaments were found assembled close to the plasma membrane, providing a framework that allowed fast changes in morphology by means of cell protrusions such as lamellipodia, filopodia, and endocytic structures. Similar observations were done by our group in other free-living amoebae such as* Acanthamoeba castellanii *[31] and* Acanthamoeba royreba* [32].

Our results show that* A. griffini* has a low* in vitro* cytopathogenicity, which is in agreement with a previous report [33] concerning the cytopathic effect of this parasite on Vero cell cultures. In interactions between MDCK cells and* A. griffini* trophozoites, the cytopathic effect on the monolayer was evident by light microscopy after 24 h. As observed by transmission electron microscopy, practically all cells exhibited a normal morphology. Nevertheless, trophozoites produced some focal injury on the cells by means of phagocytic structures of diverse size through which they engulfed portions of cells, a behavior that was previously observed in other* Acanthamoebae* such as* A. castellanii* and* A. royreba *[34, 35]. Interestingly, MDCK monolayers incubated with conditioned medium were also slightly injured, suggesting that the trophozoites produce some soluble lytic factor.

Although* A. griffini* was not able to cause an infection in the CNS in the murine GAE model, their invasive capacity was demonstrated, which correlates with a minor infection easily resolved in the patient from which it was isolated, as well as the assays with MDCK cells, which showed a cytopathic effect in less proportion than reported in other free-living amoebae.

To date,* Acanthamoeba* spp. isolates belonging to seven different genotypes (T2, T3, T4, T5, T6, T11, and T15) have been found to be associated with* Acanthamoeba* keratitis (AK) [27, 29, 36–39]. However, the most prevalent genotype in AK cases worldwide is the T4 genotype [39].

Previous studies have reported* Acanthamoeba* genotype T3 in AK cases. However this is only the report number nine of genotype T3 as a causal agent of AK and to the best of our knowledge, the second report of this genotype in a clinical case in the American continent. Other authors have isolated genotype T3 from clinical cases in a low percentage ranging from 1% to 13% in various countries including China, France, Hong Kong, Japan, Spain, Sweden, Iran, UK, and the United States [27, 29, 36, 40–44]. Furthermore, these observations are not surprising since as mentioned before genotype T4 has been reported to be the most abundant genotype in both clinical and environmental sources [39, 45], being the causative agent of around 80% of the reported infection cases worldwide [42].

Regarding environmental isolation of genotype T3, the number of isolates is also low and therefore, the low number of AK cases due to T3 could be also related to this genotype being less abundant in the environment that genotype T4 and other common genotypes in the environment genotypes such as T5 or T7. Genotype T3 has been isolated from the environment in many countries including Brazil [46], Chile [47], Egypt [17], Hong Kong [27], Iran [48], Philippines [49], Malaysia [50], and Taiwan [51]. Further epidemiological studies and pathogenicity capacity of genotype T3 isolated should be carried out worldwide in order to reach further conclusions about the status of genotype T3 as a potential pathogen in both the environment and clinical cases. Nevertheless, a recent systematic analysis of* Acanthamoeba* genotype frequency correlated with source and pathogenicity concluded that T4 is in fact the genotype most often associated with human disease, even after its abundance in the general environment is taken into account. Furthermore, T3 and T11 are closest relatives to T4 and they are the second and third most often associated with AK [52]. Further epidemiological studies on the pathogenicity capacity of genotype T3 should be performed to confirm the pathogenic status of genotype T3 in both the environment and clinical cases.

Finally, we also tested the isolate sensitivity to first line treatments currently used in AK cases. Our results showed that voriconazole inhibited the growth of* A. griffini* at very low concentrations, making it a potential first line treatment against AK cases. Other recent studies have reported similar activities of voriconazole against potentially pathogenic strains of clinical origin [23, 53] and even a successful treatment was achieved using voriconazole for the treatment of a patient suffering from AK in Spain [22].

In summary, our observations showed that the ultrastructure of* Acanthamoeba griffini *does not differ significantly from that of other* Acanthamoeba* species, Also, the* in vitro* cytopathic effect is similar to other low virulence free-living amoebae as* Acanthamoeba royreba *and is possibly the result of a low production of lytic factors by the trophozoites. Besides, this amoeba was isolated from a clinical case and belongs to genotype T3 which has clinical relevance since other pathogenic species are also included in this group. Also it was important to find that* A. griffini* is sensitive to drugs used at present in the treatment for AK. Further research with biochemical and molecular biology methods is necessary to better understand the mechanism of pathogenesis of this pathogen.
